# Economic Argument for Innovative Design From Valuing Patient-Centered Stroke Rehabilitation

**DOI:** 10.1177/19375867251327987

**Published:** 2025-04-17

**Authors:** Rhonda Kerr, Ruby Lipson-Smith, Aaron Davis, Marcus White, Mark Lam, Julie Bernhardt, Juan Pablo Saa, Tianyi Yang, the NOVELL Redesign Collaboration

**Affiliations:** 1 2720 University of Western Australia, Perth, Australia; 2 The MARCS Institute for Brain, Behaviour and Development, 6489 Western Sydney University, Westmead, NSW, Australia; 3 56369 The Florey Institute of Neuroscience and Mental Health, Heidelberg, VIC, Australia; 4 1067 University of South Australia, Adelaide, Australia; 5 Centre for Design Innovation, 3783 Swinburne University of Technology, Hawthorn, Victoria, Australia; 6 3783 Swinburne University of Technology, Hawthorn, Victoria, Australia; 7 La Trobe University, Bundoora, Victoria, Australia

**Keywords:** evidence-based design, hospital capital, hospital cost, hospital operational costs, innovation, continuous improvement, stroke, rehabilitation

## Abstract

**Purpose:** This study examines the economic benefits of innovative design in a hospital ward with the capital and operational costs and societal and government benefits. **Background:** An economic view of health care delivery options considers both the costs and benefits of an intervention for the economy, funders, and patients. Previous studies have focused on the financial costs of capital as an asset class for hospital development. **Methods:** Four hypothetical stroke rehabilitation units were designed within a larger Living Labs program (the NOVELL project). A standard stroke rehabilitation hospital ward design was compared to three alternative designs. The alternative designs expanded areas for therapy, social engagement, communal activities, and staff wellbeing, included activated corridors and enabled access to outdoor and recreational areas based on clinical evidence and expert advice. **Results:** The alternative designs are predicted to achieve A$3.3 million in savings annually for rehabilitation ward operational costs (a saving of 26%). Economy-wide benefits from the alternative designs are estimated to be A$12 million plus savings to government of between A$3.93 million and A$5.4 million per ward per annum. **Conclusions:** Adoption of innovation in design, clinical practice and evidence identification has the capacity to improve clinical effectiveness and patient outcomes. Economy wide benefits and cost improvements for health funders from the adoption of innovative design have been identified through micro- and macro-economic evaluation.

## Introduction

Nobel prize winning economists have shown the power of innovation to enhance service effectiveness and the power of appropriate capital inputs to improve the efficiency of service delivery (Schumpeter, 1942; Solow, 2005). However, the value of innovation through hospital design has been harder to prove (Ballard & Rybkowski, 2007; Berry & Kirk Hamilton, 2018). Innovation for hospital facilities to support effective clinical care has been advanced through evidence-based design and research-informed design (Berry et al., 2004; Peavey & Vander Wyst, 2017; Sadler et al., 2011). But there has been resistance to the adoption of innovative design due to perceived costs and the “willingness to pay” threshold of health officials and governments (Carvalho et al., 2022).

Research techniques and evidence have advanced enabling the design of hospital buildings to support clinical innovation more effectively. The design of a hospital building affects clinical care, staff efficiency and retention, and patient outcomes including ongoing disability and ability to work (Maben et al., 2016; Mahmood & Tayib, 2021; Saa et al., 2023). The economic impact of a hospital building should therefore consider the cost of evidence-based innovation in terms of the benefits to the economy, the health system and patients against the capital investment costs (building materials, etc.) (WHO Council on the Economics of Health for All, 2023). Replacing outmoded facilities with innovatively designed, evidence-based facilities is projected to reduce operating costs and improve patient outcomes.

However, the traditional investment view regards inpatient equipment, systems, and facilities as inert assets, separating capital investment and ward design from their clinical, operational, and broader economic impact. The value of facilitating innovative evidence-based design through appropriate capital investment in clinical pathways (Saa et al., 2023) has not previously been identified.

This paper considers the value of innovative design for hospital operational costs, costs and benefits for the patient, benefits to the economy and hospital capital costs for specific patient groups using microeconomic costing to identify costs and macro-economic measures to ascribe potential benefits.

### Patient-Centered Costing

For Australian hospitals, operational funding is patient-centered focusing on the patient, their treatment, quality measures, and improving outcomes (SCRGSP, Steering Committee for the Review of Government Service Provision, 2024). Therefore, valuing the resource inputs in this article considers the patient, activating evidence-based treatment and costing the expected outcomes (Productivity Commission, 2024a). The patient is the focus for cost measurement. The currency used is Australian dollars ($).

### The Neuroscience Optimized Virtual Environment Living Lab (NOVELL) Redesign Project

This article reports on one aspect of the Neuroscience Optimized Virtual Environments Living Lab (NOVELL) Redesign project. The aim of the NOVELL project was to optimize stroke rehabilitation (SR) care and facilities (Lipson-Smith et al., 2019). Drawing on clinical and design evidence (Blennerhassett et al., 2018; Lipson-Smith et al., 2019; Luker et al., 2015; Saa et al., 2023), we have used these defined values to redesign the models of care and the physical ward environment using innovative design for inpatient SR.

This article addresses the capital costs of four ward design options for an optimized SR unit valuing the economic benefits arising from redesigning SR wards.

### Stroke Rehabilitation in the Australian Context

Stroke costs Australia $15.7 billion over a lifetime including $5.5 billion in direct healthcare costs (Deloitte Access Economics, 2020; Kim et al., 2024). Despite survival rates for stroke improving, the annual Australian National Disability Insurance Scheme (NDIS) costs for people disabled by stroke was $1.08 billion (increasing by 38% over the year) to June 2023 (National Disability Insurance Scheme (NDIS), 2023, 2024).

Facilities for SR are rarely purpose-built, and are commonly designed similarly to acute care units, despite the increased clinical focus on activity and independence, and the prolonged length of stay (Lipson-Smith et al., 2020).

### Aim

This research aimed to assess the value of evidence-based facilities in SR for patients, the health system, and the economy. The value and cost of innovative evidence-based SR ward designs were assessed compared to a standard racetrack ward design based on the Australasian Health Facility Design Guidelines (Australasian Health Infrastructure Alliance, 2018). The assessment of costs and efficacy is patient-based to examine how clinical care is delivered for the benefit of the patient.

## Methods

The NOVELL Redesign living labs approach analyzed clinical research and expert advice to inform the SR ward designs (Bernhardt et al., 2022; Lipson-Smith et al., 2019, 2023; Saa et al., 2023). Optimal treatment pathways were developed based on the evidence and the Australasian Health Facility Guidelines (Australasian Health Infrastructure Alliance, 2018). Costs and benefits were measured at the patient level using 2020–2021 prices as external factors caused atypical price increases in subsequent years (ABS, 2023).

The following sections identify how economic benefits were costed. For hospitals national SR unit operational cost data by diagnosis group was used (IHACPA, 2023a). Expected outcomes including NDIS benefits for stroke survivors and government and societal benefits using HILDA (Household, Income and Labour Dynamics in Australia) survey data (Wilkins et al., 2024) were identified at a workshop of invited experts.

Four sets of costs are significant in our speculative SR unit design: (1) operational costs, (2) staffing costs, (3) capital costs for the construction of the unit, and (4) the cost to the individual stroke survivor and economy due to changes in disability levels and employment status. All costs are based on 2019–2020 data.

### Data Sources

#### Operational Costs

In Australia, hospital operating costs cover all hospital labor costs, supplies, pharmaceuticals, and day-to-day costs. Operating costs are paid for each episode of SR care but do not include any capital costs (IHACPA, 2023a).

SR operational costs for 2020–2021 used average length of stay data for eight patient classifications with a mean of 23 days per patient (range 11 days to 42 days per patient diagnosis group) and a mean cost per patient episode of $29,887 (range $13,428 to $55,624 per patient diagnosis group) assuming 90% occupancy (IHACPA, 2023a).

#### Staffing Costs

National average SR unit staffing costs, comprising nursing, allied health and medical salaries plus on-costs and other staff, were identified for the eight designated SR categories (IHACPA, 2023b).

#### Capital Costs

There is no national reporting of capital costs for SR or capital cost for public hospitals (AIHW, 2021, 2022; Australian Bureau of Statistics, 2022; Kerr, 2015). A costing method for capital mirroring the conventional construction process was used for a ward to be built onto an existing hospital.

### Costing Capital for Stroke Rehabilitation

Capital construction costs for a SR unit have three components:

1. physical areas directly required for SR patient care (ward areas, clinical pathway areas, clinical and non-clinical support areas, and outdoor areas)
2. areas of the hospital indirectly required to support the operation of the SR unit (hotel services, support areas, diagnostic areas, pharmacy, outpatient clinics, administration, research, training, vertical and horizontal travel areas etc.)
3. technologies and specialist equipment, information and communications technologies (ICT), and systems identified for patient care.

#### Direct Capital Costing Method for the Stroke Rehabilitation Ward

To identify total capital costs firstly, directly required areas (bed rooms, therapy spaces, corridors etc.) were identified from the architects designs and Schedules of Accommodation using the Australasian Health Facility Guidelines (AusHFG) (Australasian Health Infrastructure Alliance, 2018).

### Costs per Square Meter

Construction costs of $4,939 per square meter (or 10.76 sq ft) were applied to the directly required areas. These were from the Australian Institute of Quantity Surveyors (AIQS) cost data on subacute care specifically for bedrooms and ensuites, physiotherapy gyms, therapy areas, outdoor therapy areas and office spaces. All other areas were costed using a national average of 2019–2020 hospital cost data for the range of capital city hospitals (Rider Levett Bucknall, 2020). Detail of the costing methodology covering costing data sources is in Supplemental material. Additional costs for specific design elements and alternative forms of bedroom and bathroom configurations were included for specific designs. Time-driven Activity Based costing for health (Keel et al., 2017) was used to apportion costs.

#### Capital Costing Method for Hospital Facilities Supporting the Stroke Rehabilitation Ward

Areas indirectly required to support the SR unit were aligned with operational cost estimation methods (IHPA, 2022). Drawing from the research, the design for the model of general hospital services was developed for a contemporary Australian general hospital (Kerr, 2019). A Schedule of Accommodation was created for a typical hospital able to support Level 4–5 clinical services (Australasian Health Infrastructure Alliance, 2016). The cost per patient per year for the hospital was derived from the total cost of capital divided by the current expected lifespan of hospital units of 50 years to an annual capital cost. The annual capital cost was divided by the expected number of patients per annum to obtain the annual cost of capital per patient. Capital costs per patient were apportioned based on average length of stay. Further details of the methodology and calculations can be found in the Supplemental material.

#### Technologies and Specialist Equipment

Medical equipment, ICT systems, and their costs were included for each design. Area costs, equipment costs, and ICT costs were apportioned to the patient level based on the prevailing advice on building lifespan, and the manufacturers recommended lifespan of the systems and equipment.

The total capital cost per patient was calculated as directly and indirectly required areas per patient × costs + medical equipment × costs + ICT systems × costs.

### Analysis of Cost Used Direct Comparisons of Capital Costs to Hospital Operating Costs

#### Economic, Government, and Individual Costs

To explore the economic impact of optimizing the design of subacute rehabilitation wards for stroke survivors, co-researchers with the NOVELL Redesign network attended a workshop in April 2023. Workshop participants were targeted for their expertise (Table 1). Workshop attendees were divided into interdisciplinary groups of four-five people for initial discussion, with a facilitator present at each group. All attendees were highly familiar with the NOVELL project and understood the changes in care process and built environment design being proposed. The co-researchers were asked: (1) *What sorts of outcomes could realistically be expected if we were to construct and operate the NOVELL model?* and (2) *What would we need to measure to demonstrate the value of these outcomes?* Responses were collated and circulated to the wider NOVELL team for feedback and input. A core group of nine of the interdisciplinary workshop attendees then met regularly throughout the analysis process to provide input.

**Table 1.** The Expertise of the Workshop Attendees. Many Attendees had Dual or Previous Expertise Relevant to the Workshop Content.

Type of expertise	Number of co-researchers
Academic/Researcher	1
Architect/Designer	1
Academic/Researcher + Architect/Designer	3
Academic/Researcher + Clinician	2
Clinician	1
Clinician + Health planner	2
Health economist	1
Stroke survivor	3
Stroke survivor + Health planner	1
Stroke survivor + Clinician	2
Stroke survivor + Economist	1
Total	18

### Ward Design

**Figure 1.**
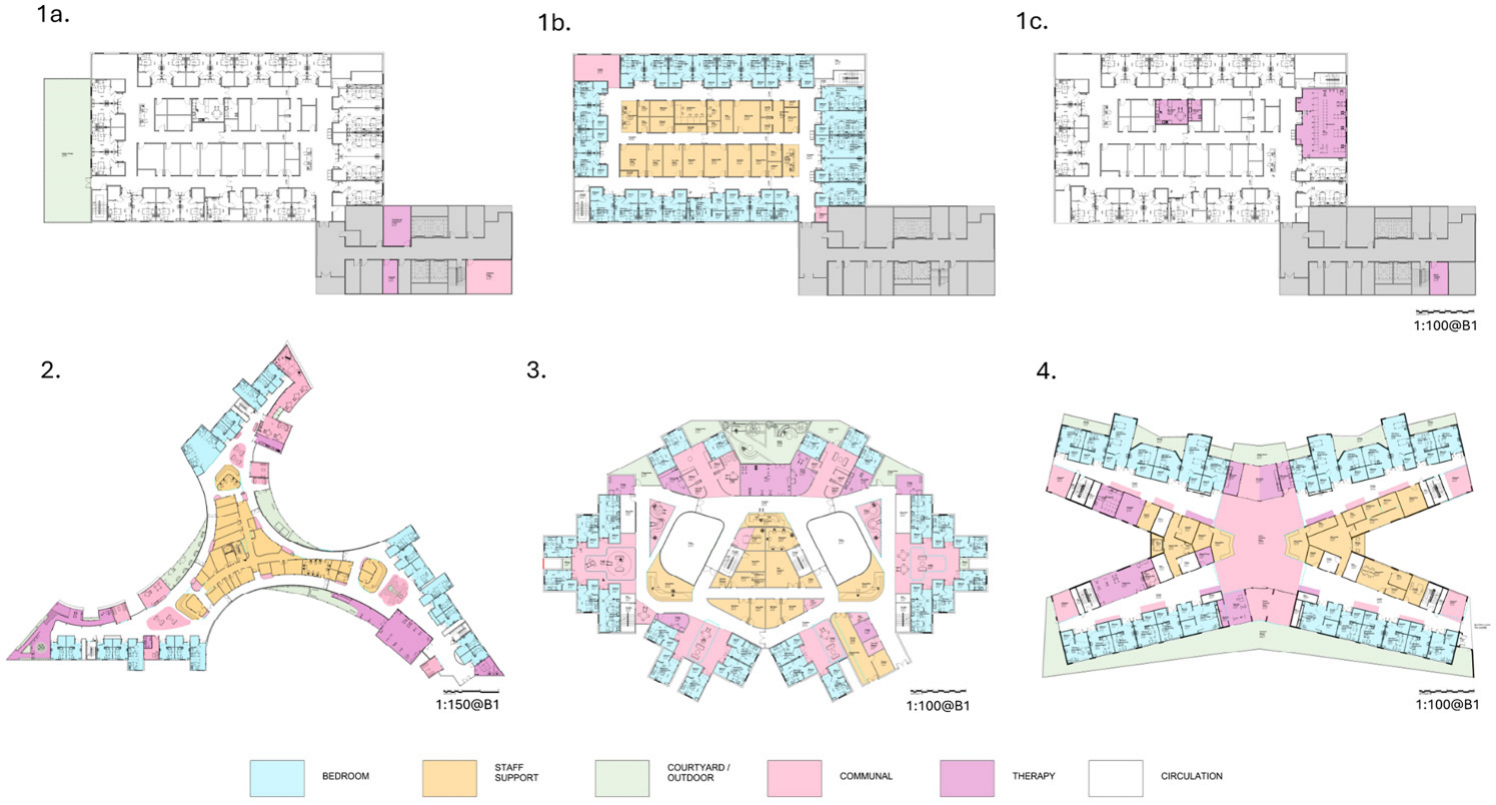
Images of (A) Racy Loop, (B) Zen, (C) Space Invader, and (D) X-wing staff, corridor, and communal areas taken from virtual reality hospital ward tours during evaluation.

**Figure 2.**
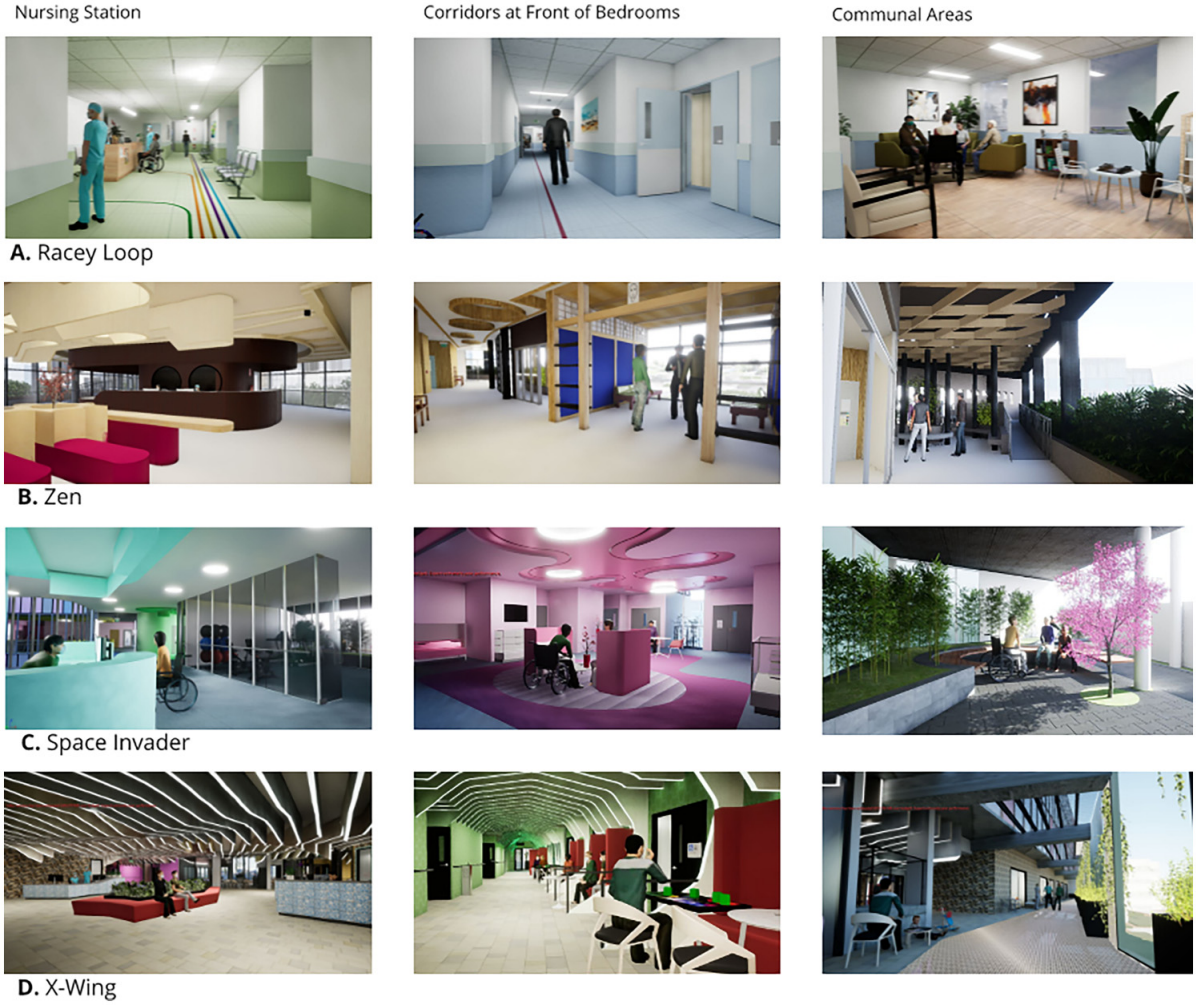
Evidence-based designs for stroke rehabilitation options for (1) Racy Loop (spread over three floors (1a, 1b, 1c), (2) Zen, (3) Space Invader, (4) X-wing.

Optimal clinical pathways were developed based on patient-centered clinical evidence translated into physical spaces incorporating stroke survivor advice, the Australasian Health Facility Guidelines (AHFG), expert clinical and architectural advice (Anåker et al., 2024; Arbel et al., 2020; Lipson-Smith et al., 2019; Saa et al., 2023). Four designs for 30 bed wards on the 6^th^ floor of a hospital are shown in Figures 1 and 2:

1. “Racy Loop” ward is a standard ward configuration is similar to many SR ward designs (Lipson-Smith et al., 2020), includes one isolation room and one patient lounge. Five rooms hold two beds sharing a bathroom. There are two nursing stations. Limited rehabilitation facilities were included in the ward area so patient and accompanying staff would be required to travel to the ground floor Allied Health Department for rehabilitation treatments. The Allied Health Department would be shared with other acute, non-acute patients, and outpatients. All ward staff areas are internal with patient rooms on the exterior of the ward. In Figure 1, the outdoor rehabilitation garden (1a, Level 5) and therapy spaces are located at other levels of the same building (1a, Level 5; 1b, Level 7).
2. “Zen” ward is in three wings of 10 beds each including rooms suitable for wheelchair users and one four-bed room with a shared bathroom. Two-bed Butterfly rooms with ensuites and balcony access can be converted to single rooms depending on patient requirements. There are five two-bed rooms with shared bathrooms. There are three nurse stations in this option and a reception area. Zen includes indoor and outdoor rehabilitation therapy areas, Activities of Daily Living (ADL) kitchen, dining and laundry areas, a telehealth room, and communal areas on the ward. Staff areas are larger in this design and allow for external views.
3. “Space Invader” ward is a more open design using central atrium areas for light and wayfinding. Bedrooms are clustered in six pods around communal areas. Therapy areas are adjacent to the bedroom pods. There are three nursing stations. Two pods have three bedrooms and two shared bathrooms. Outdoor therapy areas, two communal dining areas and a gym are included in this design. There is an isolation room.
4. “X-wing” ward features external balcony views and/or external access for all patient rooms including four two-bed rooms. Therapy areas are included in the center of the ward with two nurse stations. These include telehealth rooms, an open patient gym and a therapy gym, ADL kitchen dining and laundry areas. A central communal space unites the functions. The staff room provides access to a staff balcony.

#### Ward Design Attributes and Differences

The patient-centered designs differ significantly by the model of care, access to therapy areas, use of circulation areas, size, staff amenity, clinical areas, the types of rooms, interior design, and access to outdoor therapy, recreation and staff spaces. Additionally acoustic treatments have been included to reduce the hospital noise to allow for privacy and better sleep (Arbel et al., 2020).

##### Staffing

Optimizing the deployment of staff for clinical effectiveness is a key factor in managing operational costs in hospital units. After “Other hospital costs” (including pharmacy, imaging, supplies), ward staff comprise 68% of per patient operating costs in SR (IHACPA, 2023a). Ward nursing time is the highest cost at 30.3% (or $9,050) of operating cost, allied health staff were 17.8% of costs ($5,323), medical salaries were 11% ($3,235) of costs, and non-clinical staff costs were 10.7% ($2,755) of total costs (IHACPA, 2023a).

Staff retention has become increasingly important for effective patient care and cost management due to national and international shortages of medical, nursing and allied health staff while patient numbers increase (de Vries et al., 2023; Jarden et al., 2023; Judkins et al., 2022). There is good evidence that appropriate ward design can improve job satisfaction, efficiency, and effectiveness for clinicians (Rose et al., 2022; Sachs, 2023) and reduce clinical anxiety and stress (Iqbal & Abubakar, 2022) aiding staff retention (de Vries et al., 2023; Saba et al., 2022).

## Results

### Operational Costs

A contemporary 30 bed SR ward is calculated to cost $12.77 million per annum in operating costs based on 2020–2021 data (IHACPA, 2023a). Adopting a patient-centered costing approach, the mean operating cost was $29,887 per patient for that year.

### Model of Care

The Racy Loop design is a traditional hospital race-track design with staff centrally located but separated from therapy areas. The three alternative designs (Zen, Space Invader, and X-wing) draw on the research evidence to encourage patients to have greater agency in their rehabilitation, to move out of their rooms and be active in their times between therapy sessions, including in outdoor spaces (Luker et al., 2015; Scrivener et al., 2019).

Expanded, enticing, colorful, activated spaces have been created in the three non-traditional designs to stimulate patients visually and sensorially to facilitate social, communal and therapeutic activities (Anåker et al., 2024; Pasha & Shepley, 2024). Zen, Space Invader, and X-wing designs expand on the Racy Loop case through the inclusion of additional therapeutic environments, outdoor therapeutic and communal environments (Janssen et al., 2014), unaccompanied patient access to therapy, including telehealth extending the clinical pathway into the community, inclusion of patient-focused therapeutic technologies (Bernhardt et al., 2022), expanded staff spaces including access to outdoor spaces, diversified communal areas for patients, interiors designed to activate spaces, transformation of corridors into therapy and social or communal spaces (Kevdzija et al., 2022), and bedrooms that have the benefits of single or multiple beds.

The separation of ward and other rehabilitation areas, as in typical hospitals and Racy Loop, has been identified as a negative influence on patient activity (Blennerhassett et al., 2018). The alternative models of care allow greater patient agency, peer support and have the potential to decrease nursing and non-clinical staff time. Together these represent 41% of per patient operational costs.

In the alternative designs, decreased staff time associated with transfers of patients to other therapy and outdoor areas would deliver operating cost reductions. Where porterage transfer costs equal 50% of non-clinical staff time, there is the potential for costs per patient to be decreased by $1,377 per patient, decreasing operating costs by $638,500 per annum per ward through savings in non-clinical staff. Allied health professionals and nursing staff time would also be saved by not escorting patients to other floors of the hospital for therapy. Clinical time saved in this way could be delivered to enhanced patient care. Additionally, nursing and allied health staff savings of $1,428 per patient or 4.8% of operating costs could be found.

Access to outdoor areas over post-acute hospitalizations of between 11 and 42 days have been found to be therapeutically important (Guidolin et al., 2024; Lu et al., 2021; Ulrich et al., 2020).

Additional patient therapy time is a key outcome of the design changes which could be expected to enhance staff satisfaction and therefore staff retention, all other considerations being equal.

Similarly, access to larger staff working spaces, staff meal and break areas and outdoor environments, exterior views and natural light have been found to decrease staff stress contributing to staff satisfaction and retention (Trau et al., 2016). Additional staff spaces provide dynamic efficiency, allowing growth in future services, including virtual wards, remote monitoring and telehealth, delivered from a specialist SR unit.

### Spatial Differences

Therapy areas, patient communal areas, and activated corridors represent significant differences to the traditional approach to SR design. While Racy Loop has 419 m^2^ for these activities, Zen has 4.7 times that area (1,962 m^2^ or 21,118 sq ft), and Space Invader and X-wing have 3.5 times the area (1,487 m^2^).

The total area for Space Invader is 12.5% larger than the Racy Loop design while X-wing is 13.6% larger and Zen is 44.8% larger than Racy Loop (Table 2).

**Table 2.** Estimated Areas for the Racy Loop, Zen, Space Invader, and X-wing Wards.

Areas	Racy Loop	Zen	Space Invader	X-wing
	m^2^ (sq ft)	m^2^ (sq ft)	m^2^ (sq ft)	m^2^ (sq ft)
Patient rooms	589 (6,340)	664 (7,147)	608 (6,544)	659 (7,093)
Therapy		240 (2,583)	175 (1,883)	167 (1,797)
Outdoor therapy		356 (3,832)	203 (2,185)	474 (5,182)
Communal	44 (473)	212 (2,282)	223 (2,400)	351 (3778)
Corridors	375 (4,036)	1154 (12,422)	886 (9,536)	486 (5,231)
Staff services	130 (1,399)	236 (2,540)	194 (2,088)	172 (1,851)
Clinical support	176 (1,894)	262 (2,820)	140 (1,507)	78 (839)
Building services	75 (807)	171 (1,840)	131 (1,410)	198 (2,034)
Therapy outside ward	435 (4,682)			
Corridor to therapy	200 (2,153)			
Ward Lift foyer	51 (548)			
Outdoor recreation^a^	200 (2,153)			
Total m^2^	2275	3295	2560	2585
(Total sq ft)	(24,488)	(35,467)	(27,555)	(27,825)

a Outdoor recreation is expected to be on another floor requiring lift access and an escort.

### Estimated ward direct capital costs for four SR designs

The Racy Loop ward is estimated to cost $14.5 million to construct, including technology.

Space Invader has significant ceiling designs elements, atrium, pod lounges, dining areas, and communal spaces and is estimated to cost $16.3 million.

X-wing has additional wood elements in the corridor design and is estimated to cost $18.6 million. Acoustic adjustable ceilings for the three bed configurations in the X-wing and additional therapeutic corridor points have been included in the costs.

Zen is boldly designed with significantly more glazing, use of wood, larger staff areas and staff access to light and views and is estimated to cost $20.2 million to construct the SR ward.

Key differences between the designs are the cost of the activated corridor spaces used as casual therapy areas, outdoor therapy areas on the ward, varied patient rooms to support a diverse range of patients, and the introduction of a larger range of therapy areas on the ward.

The patient clinical pathway for Racy Loop includes lift (elevator) access to the therapy areas and outdoor spaces on another floor of the hospital at a total capital cost of $3,414,689. In the alternative designs there is a greater activation of formal and informal therapy spaces with the therapy areas of Zen, Space Invader, and X-wing estimated to cost $3,164,444 (Table 3).

**Table 3.** Estimated Direct Capital Costs for Patient Care.

Capital cost	Racy Loop	Zen	Space Invader	X-wing
Areas ($)				
Patient rooms	$5,500,000	$4,840,000	$5,720,000	$6,856,828
Therapy		$1,200,000	$875,000	$835,000
Outdoor therapy		$3,164,444	$1,804,444	$4,213,333
Communal	$217,316	$1,047,068	$1,101,397	$1,733,589
Corridors	$1,852,125	$6,839,527	$4,704,891	$2,760,407
Staff services	$541,667	$983,333	$808,333	$849,508
Clinical support	$869,264	$1,294,018	$691,460	$385,242
Building services	$370,425	$844,569	$647,009	$977,922
Therapy outside ward	$2,175,000			
Corridor to therapy	$987,800			
Ward Lift foyer	$251,889			
Outdoor recreation	$1,777,778			
Total	$14,543,263	$20,212,960	$16,352,535	$18,611,829

General lift access to the SR ward for staff, supplies, patients, and visitors is costed in indirect costs as direct construction or capital costs are defined by the patient clinical pathway.

### Indirect Capital Costs

The capital cost per patient for the elements of the hospital that support the SR unit (indirect capital costs) are estimated to be $79.42 per patient per day. As the mean length of stay in Australian SR is 23 days, total indirect capital is estimated to be $1,826 per patient. By comparison “Other hospital services” contribute $9,615 per patient or 32% of SR operating costs (IHACPA, 2023b).

### Total Capital Costs Relative to Operating Costs per Patient

Total capital construction costs for all designs are equivalent to less than 10% of operating costs per patient (Table 4). When direct and indirect capital costs are added, total capital costs per patient are between $2,507 and $2,773 per patient (Table 4) for the four designs. Differences between the capital costs of the four designs are comparatively modest representing between $85 and $265 per patient.

**Table 4.** Operating Costs and Capital Costs for Racy Loop, Zen, Space Invader, and X-wing.

Costs	Racy Loop	Zen	Space Invader	X-wing
Per patient				
Total capital cost	$2,507	$2,773	$2,592	$2,698
Operating cost	$29,887	$29,887	$29,887	$29,887
Capital to operating cost %	8.4	9.3	8.7	9
Capital:				
Variation from Racy Loop	0	$265	$85	$190
Capital variation to operating costs %	0	0.9	0.3	0.6

Capital construction costs in the Racy Loop design are equivalent to 8.4% of operating costs per patient. The alternative designs, based on the evidence of superior patient outcomes, align with between 8.7% and 9% of operating costs per patient. Put another way, the difference between the total capital costs of the designs represents between 0.3% and 0.9% of per patient operating costs. However, model of care changes associated with the designs may also reduce the operating costs. The relationships between total capital costs and operating costs per patient for each of the four designs are presented in Table 4*.*

No additional technologies or specific medical equipment needs were identified in the clinical pathways or through advice from clinicians.

#### Outcomes and Benefits

Evaluating the expected outcomes workshop participants identified the model of care options in the three alternative designs (Zen, Space Invader, and X-wing) (Yang et al., 2024). Expected outcomes and benefits included greater personal agency and stroke survivor confidence, less boredom and loneliness, positive mental health outcomes, supporting age appropriate treatment (particularly for young stroke survivors), more specific treatment options, culturally appropriate environments, supporting patients, families and carers reintegrate with work options, and ongoing support into the community for patients and families.

Benefits have been valued in dollar terms where data is available (Table 5). Benefits are categorized as hospital costs, NDIS and societal economic benefits and reducing survivor costs.

**Table 5.** Outcomes, Expected Benefits per Ward and per Patient.

Outcomes workshop	Outcomes	Expected benefit	Ward operating benefit	Per patient benefit
			$ million	$
Hospital cost benefits	Staff workflow improvements^a^^,^^b^	10% decrease average length of stay	$1.277	$2,989
	Technology enhancement	10% improvement	$1.277	$2,989
	Patients decrease dependency categories	10% change	$0.21519	$259
	Decreased rate of readmission	5% change	$0.53614	$1,263
Hospital cost savings			$3.305,334	$7,500

a Iqbal S. Hospital outdoor spaces as respite for healthcare staff during the COVID-19 pandemic. HERD 2022:15(4).

b Bourke E. It's time to rethink indoor airflow to reduce the spread of COVID-19, say experts. ABC News. 2020.

Some benefits are significant for the patient but are difficult to establish as a dollar value (e.g., an increase in personal agency for the patient during their recovery). While this is a considerable benefit and is expected to decrease length of stay and improve patient function, no dollar amount has been estimated due to gaps in the economic evidence.

### Hospital Costs

Based on the advice of the expert workshop participants, the alternative designs are predicted to achieve $3.3 million savings annually to ward operational costs of $12.77 million per annum. This is a saving of 26% on annual operating costs (Table 4). Savings are from staff workflow improvements, technological activation, increased patient activity, and decreased readmission rates (Tables 4 and 5).

In total, the expected improvements caused by evidence-based innovative design according to the workshop participants represent a decrease in per patient costs of $7,500. To achieve these outcomes, the maximum per patient capital cost is $2,773. This provides a capital cost to benefits ratio of 1 to 2.7 (Table 4).

### Disability Payment Improvements

Valuing long-term outcomes, the workshop participants predicted the effectiveness of the improved model of care in the alternative designs could be expected to decrease long-term disability for stroke survivors. The workshop participants identified that a 10% decrease in disability levels was probable from changed models of care in the alternative designs. In the words of one workshop participant: “*… if we can move ∼10% of people from more to less dependent with excellent, active rehab that delivers on guidelines and beyond then that would be a modest but achievable goal.”*

The outcome for NDIS costs would depend on the range of costs associated with each survivor, their providers cost structures, and other factors (Deloitte Access Economics, 2020). Benefits are estimated to be between $3.93 million and $5.4 million per ward (average of $4.66 million) but would require further detailed research to confirm. These estimates assume 43 patients move to discounted cost structures between 10% and 30% lower than the NDIS average payment (Deloitte Access Economics, 2020; National Disability Insurance Scheme (NDIS), 2024).

### Societal Economic Benefits

In 2020 it was estimated that the impact on wellbeing of stroke cost the Australian economy $26 billion. Productivity losses associated with reduced employment due to stroke were costed at $1.8 billion (Deloitte Access Economics, 2020).

The Wellbeing Valuation method from data gathered through the HILDA (Household, Income and Labour Dynamics in Australia) and Journeys Home surveys values moving from unemployment to employment. Experts conservatively assess that 10% more survivors would achieve employment with improved rehabilitation facilities. This would translate to benefits of $4.25 million (Wilkins et al., 2024).

With one ward of enhanced SR for all three alternative designs (Zen, Space Invader, and X-wing) it is expected a higher percentage of stroke survivors will be able to return to work with an economic value of $316,136 (Deloitte Access Economics, 2020).

Improved outcomes for survivors allow carers to return to work. If 10% of carers of patients from one ward resume their work, based on previous values, the economic benefit would be $70,649 (Deloitte Access Economics, 2020, p. 27).

Additional societal economic benefits would arise from decreased welfare dependence, lower health service use, and increased taxation but are problematic to estimate for one ward (Deloitte Access Economics, 2020).

Economic benefits are expected to be in the order of $9.3 million per annum per ward of redesigned SR. This compares to the one-off additional capital cost of between $1.8 million and $5.67 million over the standard design.

There are numerous costs met by stroke survivors (Deloitte Access Economics, 2020).

Depression is a recognized co-morbidity of stroke (Wijeratne & Sales, 2021). Workshop participants identified that survivors would have an increased sense of control in their lives building confidence and capacity from rehabilitation in the activated designs with reduced depression and anxiety after discharge. Workers with depression overall earn 30% less than their peers (Schofield et al., 2019). The HILDA (Household, Income and Labour Dynamics in Australia) study values recovery from depression at $3,320 per person or $142,760 for 10% of patients in one ward. The values used in these calculations were provided by the Australian Social Value Bank (www.asvb.com.au).

Moving from unemployment to employment using the Wellbeing Valuation method, from data gathered through the HILDA and Journeys Home surveys values, would translate to $2.633 million benefits to the economy. In total, the economic and stroke survivor benefits of one redesigned ward for SR may be in the order of $12.07 million per annum.

## Discussion

Building from the Fable hospital business case for investment in better hospitals and an improved understanding of hospital construction costs, this research considers the broader economic impact of innovative, clinically informed, research inspired innovative design through per patient costing (Sadler et al., 2008, 2009, 2011, 2021). Design activating clinical research for the benefit of patients, and improved conditions for staff, has been valued beyond the construction project, and hospital operating cost, to include the impact on patient outcomes for the economy of one ward transformed.

The purpose of a SR unit is to rehabilitate stroke survivors by delivering evidence-based therapy. The SR ward should be a place to maximize therapeutic opportunities appropriate to the patient. However, most SR wards are designed as hospital patient accommodation to inhabit between therapy sessions conducted by appointment elsewhere in the hospital.

Drawing on the clinical evidence, the NOVELL Redesign project aimed to optimize SR care aligning the facilities with the purpose of SR. Four SR ward designs were developed, assessed, and costed: one conventional hospital layout and three alternative designs responding to the research evidence.

Designing SR wards to enable evidence-based care has significant benefits for stroke survivors, the economy, the NDIS, hospital operational effectiveness, hospitals costs, and clinicians. Annual benefits of evidence-based SR design from the three alternative designs have been estimated to be $12.07 million for the Australian economy, $7.4 million for survivors, $4.66 million for the NDIS, $3.3 million for hospital operating costs, plus benefits from improved retention rates for medical, nursing and allied health staff.

The additional cost of constructing an innovative SR unit is eclipsed by the benefits and improvements for hospital operational, and staffing costs of SR. The capital cost of evidence-based innovative designs for SR was found to be equivalent to between 8.7% and 9.3% of per patient operating costs (Table 4). A traditional SR ward is estimated to have a capital cost equivalent to 8.4% of operating costs per patient.

Total capital costs for a traditional design were estimated to be $14.5 million and for the alternative evidence-based designs, to be between $16.3 and $20.2 million, excluding site and project costs (see Supplemental material). An expected 10% reduction in average length of stay represents a greater saving than the capital cost per patient for each of the three alternative designs (Table 4).

Rather than a bed-based hospital-style ward, the three alternative designs offer an activated therapeutic environment to meet the needs of stroke survivors to gain skills and experience. Three alternative ward designs, based on research findings that activated corridors, enable patients to practice therapies and tasks in preparation for returning home to the community and to work (Kevdzija et al., 2022; Wolf et al., 2009). Reducing patient-in-bed waiting time and increasing informal therapy activities permits more patients to be engaged in rehabilitation with the potential for shorter lengths of stay and enhanced outcomes, based on the literature (Janssen et al., 2014) and expert advice.

The three alternative designs support clinical pathways for patients enhancing and reinforcing the work of clinicians and avoiding boredom and loneliness identified in the literature. Technologies, including telehealth on the ward, support clinician efficiency and would permit patients to remain connected to the SR staff for telehealth outpatient support from their homes or workplaces. The traditional model involves attendance at an outpatient clinic by specialist staff, survivors, and carers. Continuity of care and extending the patient pathway back to the community is projected to save capital and operating costs for outpatient clinics, and patient travel costs.

Innovative design, the workshop participants found, improved clinical effectiveness, and provided better patient outcomes, in hospital and for the workforce. Expected reduction in disability for stroke survivors has identifiable benefits which flow through to reduced claims on the NDIS.

Innovative design has bigger impacts. Facilities can act as partners in care, enablers of formal and informal human interaction and as part of a treatment strategy (Kevdzija et al., 2022; Pilosof, 2021). Clinical research and technological innovation can be activated for the patient's benefit when there is sufficient capital to allow for change in the clinical environment.

Rather than value a SR ward as an inert hospital asset, this analysis considers the built space, and technologies as part of a dynamic treatment strategy. The utility of the treatment spaces and bedroom designs have been assessed relative to clinical evidence, expert advice, and the operational costs for delivering effective care. Outcomes encompass patient objectives and health system objectives including staff retention and the role of technology in delivering cost-effective treatment, while empowering patients in their rehabilitation journey.

The principal of patient focused costing was used to identify the utility of each model for delivering effective patient care with efficiency (Allen & Rixson, 2008; OECD, 2016). Key elements of allocative, technical, and dynamic efficiency were addressed in the costing methodology. First by specifically allocating resources to sustain clinical work and enhance patient activity. Second, aligning operating costs per patient with the capital investment per patient required to facilitate evidence-based care, allowed all the resources required to be considered at the patient level to assess technical efficiency. Third, by identifying methods to support evidence-based innovation in therapy and design enabling effective and responsive change or dynamic efficiency (Productivity Commission, 2024a).

The patient journey is the most important cost factor for hospitals. The patient clinical pathway is where most costs are incurred for the health system and the hospital (SCRGSP (Steering Committee for the Review of Government Service Provision), 2024). One type of hospital facility (and equipping) does not suit all methods of care delivery this research found. It follows therefore that the facility construction costs required to deliver clinical care are different for different types of patients due to different equipment, facility, and systems specifications. Rather than treat the hospital as a homogenous inert construction, this research considers the facilities and equipment as partners in care and avenue for continuous improvement aligned with technological and clinical change. Innovative research-based design is identified as a delivery mechanism for more cost-effective care.

Capital costs for facilities, medical equipment, and technology is often perceived as a prohibitively large cost in acute and sub-acute care (Auditor General Australia, 2023; Bernhardt et al., 2022; NSW Special Commission of Inquiry into Healthcare Funding, 2023). However, operating costs for SR are significantly larger than capital costs reflecting the cost of professional care in a hospital. Operating costs are growing at a faster rate than capital costs (IHACPA, 2023a).

Capital investment can support effective service provision with appropriate ratios of capital to operating costs to enable productivity growth (Productivity Commission, 2024b). Operating costs are adversely affected by efforts to minimize capital expenditure when designing and building health facilities (Productivity Commission, 2023). For example, rehabilitation units with required allied health facilities located away from the ward, to minimize capital costs, can adversely affect staff efficiency and effectiveness.

Replacing outmoded facilities with evidence-based facilities is projected to reduce operating costs and improve outcomes. The Racy Loop design fits a traditional model of care where activity and therapy occur on a one-to-one basis with clinical staff. In the Racy Loop model, patients need to be escorted by a clinician or staff member to another part of the hospital to receive some therapy or go to an outdoor recreation space. Clinician and staff costs represent 70% of operating costs per patient. The three alternative designs reflect a patient-focused model of care responding to the call for greater patient agency during rehabilitation with access to therapy spaces on the ward, including outdoor spaces. Therapeutic areas on the ward provide nursing and allied health staff with immediate access to appropriate facilities to assess and progress patient rehabilitation.

Additional investment in alternative evidence-based designs is calculated to increase the hours of rehabilitation activity per day per patient without increasing staffing. Better access to rehabilitation facilities and equipment under the general supervision of staff, and in common with other patients, is designed to increase patient agency, confidence, and decrease dependence on staff support.

Each of the three alternative models provides enhanced supervision of patient activity. Supervision is more subtle for the outdoor and therapy spaces in the three alternative models.

Staff have access to limited exterior views and natural light in traditional configurations, including the Racy Loop case, as staff areas are in the center of the ward (Arbel et al., 2020). The three alternative designs give staff exterior views and access to outdoor areas while on the ward. These are expected to improved staff retention, reduce stress and anxiety and sustain worker satisfaction (Saa et al., 2023; Saba et al., 2022). As the recruitment and retention of specialist staff is an issue, steps to support clinicians in a healthy enjoyable environment may provide operational cost benefits (de Vries et al., 2023).

However, SR is primarily about patient outcomes. Clinicians, researchers, and stroke survivors identified a range of probable social and economic outcomes from investment in the alternative designs including more active rehabilitation, more carers and stroke survivors returning to work, and reductions in NDIS costs per person. Stroke survivors and clinicians identified shorter lengths of stay and better patient outcomes as expected benefits of the alternative designs (Lipson-Smith et al., 2019).

A focus on the patient and their post-stroke journey identified that the effectiveness of an SR ward has significance beyond the hospital. Valuing the capital required per patient to achieve improved outcomes identified the key role specifically designed patient rooms, corridors, access to therapy spaces and communal areas have on patient outcomes. Specific facilities rather than generic design was judged to be more beneficial by the workshop participants. The workshop participants predictions aligned with the research evidence.

This study has found that the expected economic benefits exceed the one-off capital costs required to enhance SR. Capital invested to support innovative design for evidence-based SR has long-term, enduring and recurring health, economic and NDIS cost benefits and wider benefits to the economy, it was concluded.

### Strengths and Limitations

While patient-centered care is widely accepted, patient-centered costing for capital is new. The formulation of capital costing for this project provides a transparent method to enable evidence-based change in the costs for clinical facilities and equipment based on the patient and the evidence. Patient-centered capital costing has application in other areas of acute and sub-acute care.

A more detailed analysis of the three alternative designs for staff movement, staff preferences, clinical service delivery, the optimal size of outdoor areas, and operating costs per patient is necessary to identify the best long-term design option.

While the outcomes and benefits were carefully assessed using a range of verification tools, the outcomes are theoretical. Further research is required on a cohort of stroke survivor outcomes of an SR unit built to these principles to accurately measure results. Further research is also required to build tools clinicians and hospital administrators can use to evaluate the wider benefits of innovative, evidence-based designs.

## Conclusions

Investment in innovative design provides on-going benefits to the economy, patients, and the health system that significantly outweighs the capital cost. Capital investment aligned to the unit purpose, patients, clinical standards, and clinical evidence can enable more innovative designs to support effective and efficient clinical care.

Aligning innovative design and appropriate capital investment to patient outcomes, based on rigorous clinical evidence, has been found to have significant benefits for stroke survivors, clinicians, hospital costs, carers, the disability payments program, and for the economy. Purposeful research combined with the advice of experts with lived experience, delivered rich data to enable more appropriate, and responsive designs for SR delivery.

## Supplemental Material

sj-docx-1-her-10.1177_19375867251327987 - Supplemental material for Economic Argument for Innovative Design From Valuing Patient-Centered Stroke Rehabilitation Supplemental material, sj-docx-1-her-10.1177_19375867251327987 for Economic Argument for Innovative Design From Valuing Patient-Centered Stroke Rehabilitation by Rhonda Kerr, BA(Econs), PhD, Ruby Lipson-Smith, BA, BSc (Hons)PhD, Aaron Davis, B Mus, B Arch S, M Arch, MSD, PhD, Marcus White, B Arch, PhD, Mark Lam, BPD, BSc(Hons)M Arch, PhD, Julie Bernhardt, BAppSci (Physio), PhD, Juan Pablo Saa, MPH, PhD, OTD, Tianyi Yang, M Arch, and the NOVELL Redesign Collaboration in HERD: Health Environments Research & Design Journal

sj-docx-2-her-10.1177_19375867251327987 - Supplemental material for Economic Argument for Innovative Design From Valuing Patient-Centered Stroke Rehabilitation Supplemental material, sj-docx-2-her-10.1177_19375867251327987 for Economic Argument for Innovative Design From Valuing Patient-Centered Stroke Rehabilitation by Rhonda Kerr, BA(Econs), PhD, Ruby Lipson-Smith, BA, BSc (Hons)PhD, Aaron Davis, B Mus, B Arch S, M Arch, MSD, PhD, Marcus White, B Arch, PhD, Mark Lam, BPD, BSc(Hons)M Arch, PhD, Julie Bernhardt, BAppSci (Physio), PhD, Juan Pablo Saa, MPH, PhD, OTD, Tianyi Yang, M Arch, and the NOVELL Redesign Collaboration in HERD: Health Environments Research & Design Journal

## References

[bibr1-19375867251327987] ABS. (2023). *Producer Price Indexes, Australia*. https://www.abs.gov.au/statistics/economy/price-indexes-and-inflation/producer-price-indexes-australia/jun-2023

[bibr2-19375867251327987] AIHW. (2021). *Health Expenditure Australia 2019–20* Canberra. https://www.aihw.gov.au/getmedia/f1284c51-e5b7-4059-a9e3-c6fe061fecdc/Health-expenditure-Australia-2019-20.pdf.aspx?inline=true

[bibr3-19375867251327987] AIHW. (2022). *Hospital Resources 2020–21*. Canberra. https://www.aihw.gov.au/reports-data/myhospitals/sectors/admitted-patients

[bibr4-19375867251327987] Allen D. Rixson L. (2008). How has the impact of ‘care pathway technologies’ on service integration in stroke care been measured and what is the strength of the evidence to support their effectiveness in this respect? International Journal of Evidence Based Healthcare, 6(1), 78–110. 10.1111/j.1744-1609.2007.00098.x 21631815

[bibr5-19375867251327987] Anåker A. Kevdzija M. Elf M. (2024). Enriched environments in stroke units: Defining characteristics and limitations. HERD: Health Environments Research & Design Journal, 17(2), 344–359. 10.1177/19375867231224972 38494920 PMC11080395

[bibr6-19375867251327987] Arbel I. Ye B. Mihailidis A. (2020). Stroke Patients’ experiences in an adaptive healing room in a stroke rehabilitation unit. HERD: Health Environments Research & Design Journal, 13(2), 170–185. 10.1177/1937586719879060 31631699

[bibr7-19375867251327987] Auditor General Australia. (2023). *Administration of the Community Health and Hospitals Program*. https://www.anao.gov.au/sites/default/files/2023-06/Auditor-General_Report_2022-23_31.pdf

[bibr8-19375867251327987] Australasian Health Infrastructure Alliance. (2016). *Australasian Health Facility Guidelines Part B - Health Facility Briefing and Planning 0080 - General Requirements*. Sydney. https://aushfg-prod-com-au.s3.amazonaws.com/download/Part%20B.0080_5.pdf

[bibr9-19375867251327987] Australasian Health Infrastructure Alliance. (2018). *Australasian Health Facility Guidelines Part B - Health Facility Briefing and Planning 0610 - Rehabilitation Inpatient Unit*. https://aushfg-prod-com-au.s3.amazonaws.com/HPU_B.0610_2_0.pdf

[bibr10-19375867251327987] Australasian Health Infrastructure Alliance. (2022). *Part B - Health Facility Briefing and Planning 0700 – Logistics / Back of House Services*. https://aushfg-prod-com-au.s3.amazonaws.com/HPU_B.0700_2.pdf

[bibr11-19375867251327987] Australasian Health Infrastructure Alliance. (2023). *Part B - Health Facility Briefing and Planning 0430 – Front of House Unit*. https://aushfg-prod-com-au.s3.amazonaws.com/HPU_B.0430_7.pdf

[bibr12-19375867251327987] Australian Bureau of Statistics. (2022). *Understanding the Different Approaches to Reporting Health Expenditure in Australia*. https://www.abs.gov.au/statistics/research/understanding-different-approaches-reporting-health-expenditure-australia

[bibr13-19375867251327987] Ballard G. Rybkowski Z. (2007). The evidence based design literature review and its potential implications for capital budgeting of healthcare facilities. U. H. R. a. E. Trust.

[bibr14-19375867251327987] Bernhardt J. Lipson-Smith R. Davis A. White M. Zeeman H. Pitt N. Shannon M. Crotty M. Churilov L. Elf M. (2022). Why hospital design matters: A narrative review of built environments research relevant to stroke care. International Journal of Stroke, 17, 370–377. https://doi.org/10.1177/17474930211042485 34427477 10.1177/17474930211042485 PMC8969212

[bibr15-19375867251327987] Berry L. L. Kirk Hamilton D. (2018). How to build a better, safer, more welcoming hospital. The Conversation, *3 July,* 2018. Retrieved July 9, 2018, from https://theconversation.com/how-to-build-a-better-safer-more-welcoming-hospital-98532

[bibr16-19375867251327987] Berry L. L. Parker D. Coile R. C. Hamilton K. D. O’Neill D. Sadler B. L. (2004). The business case for better buildings. Frontiers of Health Services Management, 21(1), 3–24. 10.1097/01974520-200407000-00002 15469120

[bibr17-19375867251327987] Blennerhassett J. M. Borschmann K. N. Lipson-Smith R. A. Bernhardt J. (2018). Behavioral mapping of patient activity to explore the built environment during rehabilitation. HERD: Health Environments Research & Design Journal, 11(3), 109–123. 10.1177/1937586718758444 29564923

[bibr18-19375867251327987] Boluijt P. Hinkema M . (2005). *Future hospitals: competitive and healing*. Netherlands Board for Hospital Institutions, Utrecht.

[bibr19-19375867251327987] Carvalho N. Sousa T. V. Mizdrak A. Jones A. Wilson N. Blakely T. (2022). Comparing health gains, costs and cost-effectiveness of 100 s of interventions in Australia and New Zealand: An online interactive league table. Popul Health Metrics, 20, 17. 10.1186/s12963-022-00294-3 PMC9327210 35897104

[bibr20-19375867251327987] Deloitte Access Economics. (2020). *The economic impact of stroke in Australia,* 2020.

[bibr21-19375867251327987] Department of Health WA. (2013). *WA Health Clinical Services Framework 2014–*2024. http://ww2.health.wa.gov.au/∼/media/Files/Corporate/Reports%20and%20publications/Clinical%20Services%20Framework/Clinical_Framework_2014-2024.ashx

[bibr22-19375867251327987] de Vries N. Boone A. Godderis L. Bouman J. Szemik S. Matranga D. de Winter P. (2023). The race to retain healthcare workers: A systematic review on factors that impact retention of nurses and physicians in hospitals. INQUIRY: The Journal of Health Care Organization, Provision, and Financing, 60, 00469580231159318. 10.1177/00469580231159318 PMC10014988 36912131

[bibr23-19375867251327987] Guidolin K. Jung F. Hunter S. Yan H. Englesakis M. Verderber S. Chadi S. Quereshy F. (2024). The influence of exposure to nature on inpatient hospital stays: A scoping review. HERD: Health Environments Research & Design Journal, 17, 360–375. 10.1177/19375867231221559 38288612 PMC11080386

[bibr24-19375867251327987] IHACPA. (2023a). *National Hospital Cost Data Collection: Public Sector Report – 2020-21 Financial Year*. https://www.ihacpa.gov.au/sites/default/files/2023-06/national_hospital_cost_data_collection_report_public_sector_2020-21_0.pdf https://www.ihacpa.gov.au/resources/national-hospital-cost-data-collection-nhcdc-public-sector-report-2020-21

[bibr25-19375867251327987] IHACPA. (2023b). *National Hospital Cost Data Collection: Public Sector Report, 2020-21 Financial Year — June* 2023. Sydney. https://www.ihacpa.gov.au/resources/national-hospital-cost-data-collection-nhcdc-public-sector-report-2020-21

[bibr26-19375867251327987] IHPA. (2022). *National Hospital Cost Data Collection Public Sector, Round 24 (financial year 2019–20)*. https://www.ihpa.gov.au/publications/national-hospital-cost-data-collection-public-sector-round-24-financial-year-2019-20

[bibr27-19375867251327987] Iqbal S. A. Abubakar I. R. (2022). Hospital outdoor spaces as respite areas for healthcare staff during the COVID-19 pandemic. HERD: Health Environments Research & Design Journal, 15(4), 343–353. 10.1177/19375867221111530 35831995

[bibr28-19375867251327987] Janssen H. Ada L. Bernhardt J. McElduff P. Pollack M. Nilsson M. Spratt N. J. (2014). An enriched environment increases activity in stroke patients undergoing rehabilitation in a mixed rehabilitation unit: A pilot non-randomized controlled trial. Disabil Rehabil, 36(3), 255–262. 10.3109/09638288.2013.788218 23627534

[bibr29-19375867251327987] Jarden R. J. Scott S. Rickard N. Long K. Burke S. Morrison M. Mills L. Barker E. Sharma K. Twomey B. (2023). Factors contributing to nurse resignation during COVID-19: A qualitative descriptive study. Journal of Advanced Nursing, 79, 2484–2501. 10.1111/jan.15596 36805610

[bibr30-19375867251327987] Judkins S. Kinder S. Govindasamy L. Toogood G. Davis J. (2022). The other long COVID: impacts on health systems and clinicians. *MJA Insight*, *31 January,* 2022. https://insightplus.mja.com.au/2022/3/the-other-long-covid-impacts-on-health-systems-and-clinicians/

[bibr31-19375867251327987] Keel G. Savage C. Rafiq M. Mazzocato P. (2017). Time-driven activity-based costing in health care: A systematic review of the literature. Health Policy, 121, 755–763. 10.1016/j.healthpol.2017.04.013 28535996

[bibr32-19375867251327987] Kerr R. (2015). Concerns about the Australian institute of health and welfare’s information on capital investment for healthcare. Australian Health Review, 39(4), 453–454. 10.1071/AH14180 25844879

[bibr33-19375867251327987] Kerr R. (2019). Can diagnosis-based capital allocation facilitate more appropriate, sustainable and innovative acute care? Curtin University]. Curtin University Theses. http://hdl.handle.net/20.500.11937/77705

[bibr34-19375867251327987] Kevdzija M. Bozovic-Stamenovic R. Marquardt G. (2022). Stroke Patients’ free-time activities and spatial preferences during inpatient recovery in rehabilitation centers. HERD: Health Environments Research & Design Journal, 15(4), 96–113. 10.1177/19375867221113054 PMC9523820 35850529

[bibr35-19375867251327987] Kim J. Neville E. Dalli L. Zomer E. Birhanu M. Purvis T. Olaiya M. T. Talic S. Kilkenny M. F. Cadilhac D. A. (2024). Economic Impact of Stroke 2024.

[bibr36-19375867251327987] Lipson-Smith R. Churilov L. Newton C. Zeeman H. Bernhardt J. (2019). Framework for designing inpatient stroke rehabilitation facilities: A new approach using interdisciplinary value-focused thinking. HERD: Health Environments Research & Design Journal, 12(4), 142–158. 10.1177/1937586719831450 30799632 PMC6745610

[bibr37-19375867251327987] Lipson-Smith R. Zeeman H. Bernhardt J. (2020). What’s in a building? A descriptive survey of adult inpatient rehabilitation facility buildings in Victoria, Australia. Archives of Rehabilitation Research and Clinical Translation, 2(1), 100040. 10.1016/j.arrct.2020.100040 33543069 PMC7853350

[bibr38-19375867251327987] Lipson-Smith R. Zeeman H. Muns L. Jeddi F. Simondson J. Bernhardt J. (2023). The role of the physical environment in stroke recovery: Evidence-based design principles from a mixed-methods multiple case study. PLoS ONE, 18(6), e0280690. 10.1371/journal.pone.0280690 PMC10256226 37294748

[bibr39-19375867251327987] Lu S. Zhao Y. Liu J. Xu F. Wang Z. (2021). Effectiveness of horticultural therapy in people with schizophrenia: A systematic review and meta-analysis. Effectiveness of Horticultural Therapy in People with Schizophrenia: A Systematic Review and Meta-Analysis, 18(3), 964. 10.3390/ijerph18030964 PMC7908324 33499390

[bibr40-19375867251327987] Luker J. Lynch E. Bernhardsson S. Bennett L. Bernhardt J. (2015). Stroke survivors’ experiences of physical rehabilitation: A systematic review of qualitative studies. Archives of Physical Medicine and Rehabilitation, 96(9), 1698–1708. 10.1016/j.apmr.2015.03.017 25847387

[bibr41-19375867251327987] Maben J. Griffiths P. Penfold C. Simons M. Anderson J. E. Robert G. Pizzo E. Hughes J. Murrells T. Barlow J. (2016). One size fits all? Mixed methods evaluation of the impact of 100% single-room accommodation on staff and patient experience, safety and costs. BMJ Quality & Safety, 25, 241–256. http://qualitysafety.bmj.com/content/25/4/241.abstract https://doi.org/10.1136/bmjqs-2015-004265 10.1136/bmjqs-2015-004265 PMC4819646 26408568

[bibr42-19375867251327987] Mahmood F. J. Tayib A. Y. (2021). Healing environment correlated with patients’ psychological comfort: Post-occupancy evaluation of general hospitals. Indoor and Built Environment, 30(4), 180–194. 10.1177/1420326X19888005

[bibr43-19375867251327987] National Disability Insurance Scheme (NDIS). (2023). Stroke data to 31 March 2023- 30 September 2023. In S. d. t. M. S. Payments (Ed.), *Stroke data to 31 March 2023 (XLSX 39KB)*. https://data.ndis.gov.au/reports-and-analyses/participant-dashboards/stroke

[bibr44-19375867251327987] National Disability Insurance Scheme (NDIS). (2024). *Stroke data and dashboard to 30 June* 2023. https://data.ndis.gov.au/reports-and-analyses/participant-dashboards/stroke

[bibr45-19375867251327987] Netherlands Board for Healthcare Institutions. (2007). Building differentiation of hospitals. Layers Approach Report No. 611, Utrecht.

[bibr46-19375867251327987] NSW Health. (2016). NSW Health Guide to the Role Delineation of Clinical Services. In. http://www.health.nsw.gov.au/services/Publications/role-delineation-of-clinical-services.PDF: NSW Health Health System Planning and Investment Branch in conjunction with the Guide to the Role Delineation of Clinical Services Reference Group

[bibr47-19375867251327987] NSW Special Commission of Inquiry into Healthcare Funding. (2023). *Transcript Day 3 Ms Willcox CEO NSW Health*. https://healthcarefunding.specialcommission.nsw.gov.au/documents/

[bibr48-19375867251327987] OECD. (2016). *Scoping paper on health system efficiency measurement (cooperation between the OECD and EC in promoting efficiency in health care)*. Brussells. https://www.oecd.org/health/health-systems/Scoping-Paper-Measuring-efficiency-in-health-system.pdf

[bibr49-19375867251327987] Pasha S. Shepley M. (2024). A structured literature review on the research and design of rehabilitation environments. HERD: Health Environments Research & Design Journal, 17(3), 354–371. 10.1177/19375867241248604 38742748

[bibr50-19375867251327987] Peavey E. Vander Wyst K. B. (2017). Evidence-Based design and research-informed design: What’s the difference? Conceptual definitions and comparative analysis. HERD: Health Environments Research & Design Journal, 10(5), 143–156. 10.1177/1937586717697683 28349729

[bibr51-19375867251327987] Pilosof N. P. (2021). Building for change: Comparative case study of hospital architecture. Health Environments Research & Design Journal, 14(1), 47–60. 10.1177/1937586720927026 32539464 PMC7934159

[bibr52-19375867251327987] Productivity Commission. (2023). *5-year Productivity Inquiry: Advancing Prosperity Inquiry report – volume 1*. https://www.pc.gov.au/inquiries/completed/productivity/report/productivity-advancing-prosperity-all-volumes.pdf

[bibr53-19375867251327987] Productivity Commission. (2024a). *Advances in measuring healthcare productivity*. Canberra, https://www.pc.gov.au/research/completed/measuring-healthcare-productivity/measuring-healthcare-productivity.pdf

[bibr54-19375867251327987] Productivity Commission. (2024b). *Annual productivity bulletin* 2024. https://www.pc.gov.au/ongoing/productivity-insights/bulletins/bulletin-2024/productivity-bulletin-2024.pdf

[bibr55-19375867251327987] Queensland Department of Health. (2010). *Guide to health service planning*. http://www.capital.dhs.vic.gov.au/Assets/Files/Q%20Guide%20to%20HSP_WEB%20vers_19Feb2010.pdf

[bibr56-19375867251327987] Rider Levett Bucknall. (2020). Riders Digest 2020 48th Edition.

[bibr57-19375867251327987] Rose S. J. Waggener L. Kiely S. C. Hedge A. (2022). Postoccupancy evaluation of a neighborhood concept redesign of an acute care nursing unit in a planetree hospital. HERD: Health Environments Research & Design Journal, 15(3), 171–192. 10.1177/19375867221091318 35389291

[bibr58-19375867251327987] Saa J. P. Lipson-Smith R. White M. Davis A. Yang T. Wilde J. Blackburn M. Churilov L. Bernhardt J. (2023). Stroke inpatient rehabilitation environments: Aligning building construction and clinical practice guidelines through care process mapping. Stroke, 54, 2946–2957. 10.1161/STROKEAHA.123.044216 37846565

[bibr59-19375867251327987] Saba J. Hullinger R. McCoy T. H. (2022). Designing Hospitals that Promote Staff Wellbeing. Harvard Business Review.https://doi.org/https://hbr.org/2022/06/designing-hospitals-that-promote-staff-wellbeing

[bibr60-19375867251327987] Sachs N. A. (2023). Caring for caregivers: Access to nature for healthcare staff. HERD: Health Environments Research & Design Journal, 16(4), 206–212. 10.1177/19375867231194780 37621161

[bibr61-19375867251327987] Sadler B. Berry L. Guenther R. Hamilton K. Hessler F. Merritt C. Parker D . (2011). Fable hospital 2.0: The business case for building better health care facilities. The Hastings Center Report, 41(1), 13–23. 10.1002/j.1552-146x.2011.tb00093.x 21329099

[bibr62-19375867251327987] Sadler B. L. DuBose J. Zimring C. (2008). The business case for building better hospitals through evidence-based design. Health Environments Research and Design Journal, 1(3), 22–39. 10.1177/193758670800100304 21161906

[bibr63-19375867251327987] Sadler B. Joseph A. Keller A. Rostenberg B. (2009). Using Evidence-Based Environmental Design to Enhance Safety and Quality. Institute for Healthcare Improvement.

[bibr64-19375867251327987] Schofield D. Cunich, M. Shrestha R. Tanton R. Veerman L. Kelly S. Passey M. (2019). Indirect costs of depression and other mental and behavioural disorders for Australia from 2015 to 2030. BJPsych Open, 5(5), e40. https://doi.org/10.1192/bjo.2019.26 10.1192/bjo.2019.26 PMC6520529 31530305

[bibr65-19375867251327987] Schumpeter J. (1942). Creative destruction. In Capitalism, socialism and democracy (pp. 82–85). Harpers.

[bibr66-19375867251327987] SCRGSP (Steering Committee for the Review of Government Service Provision). (2024). *Report on Government Services* 2024. Canberra. https://www.pc.gov.au/ongoing/report-on-government-services/2024/health/Rogs-2024-parte-overview-and-sections.pdf

[bibr67-19375867251327987] Scrivener K. Pocovi N. Jones T. Dean B. Gallagher S. Henrisson W. Thorburn M. Dean C . (2019). Observations of activity levels in a purpose-built, inpatient, rehabilitation facility. HERD: Health Environments Research & Design Journal, 12(4), 26–38. 10.1177/1937586718823519 30727762

[bibr68-19375867251327987] Sdino L. Brambilla A. Dell’Ovo M. Sdino B. Capolongo S. (2021). Hospital construction cost affecting their lifecycle: An Italian overview. Healthcare, 9(7), 888. https://doi.org/10.3390/healthcare9070888 34356266 10.3390/healthcare9070888 PMC8303202

[bibr69-19375867251327987] Solow R. M. (2005). Flexibility and endogenous innovation. The Journal of Technology Transfer, 30(1–2), 11–15.

[bibr71-19375867251327987] Trau D. Keenan K. Goforth M. Large V. (2016). Nature contacts: Employee wellness in healthcare. HERD: Health Environments Research & Design Journal, 9(3), 47–62. 10.1177/1937586715613585 26578539

[bibr72-19375867251327987] Ulrich R. Cordoza M. Gardiner S. Manulik B. Fitzpatrick P. Hazen T. Perkins R. S. (2020). ICU Patient family stress recovery during breaks in a hospital garden and indoor environments. HERD: Health Environments Research & Design Journal, 13(2), 83–102. 10.1177/1937586719867157 31390887

[bibr73-19375867251327987] Victorian Department of Health. (2013). *Benchmarking-Service Planning Data Input*. Melbourne. http://www.capital.health.vic.gov.au/capdev/ProjectProposals/Benchmarking/ServicePlanDataInput/

[bibr74-19375867251327987] WHO Council on the Economics of Health for All. (2023). *Health for All – transforming economies to deliver what matters: final report of the WHO Council on the Economics of Health for All* .

[bibr75-19375867251327987] Wijeratne T. Sales C. (2021). Understanding why post-stroke depression may be the norm rather than the exception: The anatomical and neuroinflammatory correlates of post-stroke depression. Journal of Clinical Medicine, 10(8), 1674. 10.3390/jcm10081674 33919670 PMC8069768

[bibr76-19375867251327987] Wilkins R. Vera-Toscano E. Botha F. (2024). The Household, Income and Labour Dynamics in Australia Survey: Selected Findings from Waves 1 to 21.

[bibr77-19375867251327987] Wolf T. J. Baum C Connor L. T. (2009). Changing face of stroke: Implications for occupational therapy practice. American Journal of Occupational Therapy, 63, 621–625. 10.5014/ajot.63.5.621 PMC2862359 19785261

[bibr78-19375867251327987] Yang T. White M. Lipson-Smith R. Shannon M. M. Latifi M. (2024). Design decision support for healthcare architecture: A VR-integrated approach for measuring user perception. Buildings, 14, 797. https://doi.org/10.3390/buildings14030797

