# Supplementary material for: Economic Argument for Innovative Design From Valuing Patient-Centered Stroke Rehabilitation
Source: HERD. 2025 Apr 17;18(3):95–113. doi: 10.1177/19375867251327987 (PMC12340140; doi:10.1177/19375867251327987)
Supplement: sj-docx-1-her-10.1177_19375867251327987 - Supplemental material for Economic Argument for Innovative Design From Valuing Patient-Centered Stroke Rehabilitation [file sj-docx-1-her-10.1177_19375867251327987.docx]

**Supplementary material**

**Costing data source**

There is no national data published on the capital cost for public hospitals (AIHW, 2021, 2022; Australian Bureau of Statistics, 2022; Kerr, 2015). To overcome the data issue three options for area costing data were identified and reviewed:

1. Published high level hospital and nursing home area costings from reputable firms of Quantity Surveyors.

*Advantages*- readily available. *Disadvantage*. Costings are by major city and at high level for acute hospitals rather than bespoke rehabilitation units.

1. The Australian Institute of Quantity Surveyors (AIQS)

*Advantages-* data specific to room type for subacute facilities in 2019-20 dollars sourced from the national body. *Disadvantages*- not independently verifiable

1. Advice from Swinburne faculty on specific building product costing.

**Cost data**

It was concluded that the AIQS sourced data would be the strongest for use in specified areas with national costing data applied to common areas and advice from Swinburne faculty for specific design features.

AIQS supplied cost data on subacute care for 2021 was used specifically for bedrooms and ensuites, physiotherapy gyms, therapy areas, outdoor therapy areas and office spaces. All other areas were costed using a national average of 2019-20 hospital cost data for the range of capital city hospitals with a 55-80m² Gross Floor Area per bed (Rider Levett Bucknall, 2020). Using this index, the national average cost per square meter was calculated to be $4,939.00.

**Indirect Costs required to support the SR unit**

An indirect Areas Cost model was required to cover the range of services expected to support a SR unit. Drawing from the research, the design for the model of general hospital services was developed for a contemporary Australian Level 4-5 hospital (Kerr, 2019). Design of the model drew on studies of capital allocative effectiveness from the Netherlands and the USA (Boluijt, 2005; Netherlands Board for Healthcare Institutions, 2007; Sadler, 2011). First, hospital departments were identified then data sources for hospital department areas were determined. Three Australian sources were used to identify relevant departments, relative bed numbers and indicative areas, the Australasian Health Facility Guideline list of Health Planning Units and non-clinical services (Australasian Health Infrastructure Alliance, 2018, 2022, 2023), Role Delineation statements and planning guidelines for hospital services (Department of Health WA, 2013; NSW Health, 2016; Queensland Department of Health, 2010; Victorian Department of Health, 2013) and Area Schedules for three Australian hospitals.

Validation of the model area schedule was made by an expert panel comprising senior hospital executives and hospital architects (Kerr, 2019).

Areas not required to support SR such as obstetrics and paediatrics have been removed from indirect areas. For models where rehabilitation gyms and Activity of Daily Living (ADL) areas are on the SR ward, these areas have been removed from the total indirect areas required to support the SR unit and have been added to the costs of the ward areas.

Exclusions from capital cost estimation

Maintenance costs are not included. Indirect capital costing does not include vehicles, roads, parking, legal and professional costs, land acquisition, project costs, or site mitigation costs as these vary significantly between projects. Costings assume a level green fields site with water, sewerage and electricity connections. It is assumed that the Stroke Rehabilitation Unit (SRU) will be above the 4^th^ floor of a level 4-5 general hospital with lift access to the unit, as is typical of rehabilitation facilities in Australia (Lipson-Smith, 2020).

Research and teaching spaces are assumed to be elsewhere on the hospital campus. Costings are for full building costs including building systems and large clinical equipment covered by Construction Contract Schedule 1 and 2 but not Schedule 3 furniture and fittings or Schedule 4 client installed items. All costs exclude GST.
