# Supplementary material for: Economic Argument for Innovative Design From Valuing Patient-Centered Stroke Rehabilitation
Source: HERD. 2025 Apr 17;18(3):95–113. doi: 10.1177/19375867251327987 (PMC12340140; doi:10.1177/19375867251327987)
Supplement: sj-docx-2-her-10.1177_19375867251327987 - Supplemental material for Economic Argument for Innovative Design From Valuing Patient-Centered Stroke Rehabilitation [file sj-docx-2-her-10.1177_19375867251327987.docx]

Economic argument for innovative design

from valuing patient-centered

stroke rehabilitation

**Implications for practice**

It shows fiscal responsibility to invest in innovative design to enable evidence-led clinical innovation.

The alignment of innovative design and the Living Labs methodology has operational and economic benefits significantly exceeding the capital costs.

This research demonstrates an alternative methodology for evaluating the costs and benefits of design options so that clinical effectiveness can be enabled through evidence-based, technologically informed patient clinical pathways.

Activating inpatient facilities to meet the specific requirements of clinical effectiveness for specific patient diagnosis groups was more cost effective than the standard or traditional design option.
